# Designing Vaccines for the Twenty-First Century Society

**DOI:** 10.3389/fimmu.2014.00012

**Published:** 2014-01-23

**Authors:** Oretta Finco, Rino Rappuoli

**Affiliations:** ^1^Research Center, Novartis Vaccines and Diagnostics, Siena, Italy

**Keywords:** vaccination, glyco-conjugate, technologies, vaccine design, structural vaccinology

## Abstract

The history of vaccination clearly demonstrates that vaccines have been highly successful in preventing infectious diseases, reducing significantly the incidence of childhood diseases and mortality. However, many infections are still not preventable with the currently available vaccines and they represent a major cause of mortality worldwide. In the twenty-first century, the innovation brought by novel technologies in antigen discovery and formulation together with a deeper knowledge of the human immune responses are paving the way for the development of new vaccines. Final goal will be to rationally design effective vaccines where conventional approaches have failed.

## Introduction

In the last century, vaccines demonstrated to be a successful and effective medical intervention representing one of the most important applications of immunology to prevent infectious diseases. A landmark in the history of immunology is the experiment conducted by Edward Jenner in 1796, when he demonstrated that inoculation with pus from cowpox lesions was conferring protection against smallpox infection (1) providing an innovative contribution to immunization and the ultimate eradication of smallpox (2). Smallpox was one of the most severe human diseases, responsible only in Europe for the death of more than 400,000 people per year. In 1979, smallpox was eradicated through a global vaccine administration campaign. Jenner’s work was further refined by Louis Pasteur, who artificially attenuated viruses for use in vaccines and in 1885 developed the first rabies human vaccine. He brought a breakthrough in the prevention and treatment of infectious diseases by establishing the basis of vaccinology, meaning the principle of isolation, inactivation, and administration of disease causing pathogens. The Pasteur’s principles have allowed the development of “first generation” vaccines based on whole microorganism killed or live-attenuated (e.g., *Bacillus Calmette Guerin* BCG, plague, pertussis, and smallpox) (3, 4).

In the second half of twentieth century improvements and innovation in mammalian cell culture technology led to the growth of viruses and development of live attenuated “second generation” vaccines such as polio (Sabin oral), measles, rubella, mumps, and varicella. More recently, the use of inactivated polio vaccine (Salk type) together with oral vaccine has almost eradicated polio from the world thanks to global vaccination (5). In developed countries national immunization programs have drastically reduced most of the viral and bacterial infections that traditionally affected children. In May 2012, the 194 Member States of the World Health Organization Assembly endorsed the global vaccine action plan (GVAP) with the vision of delivering universal access to immunization, with at least 2–3 million lives saved per year worldwide (http://www.who.int/immunization/global_vaccine_action_plan/). Although traditionally developed vaccines have been in the last century of unquestionable value, saving more than 700 million cases of disease and more than 150 million deaths, the conventional methods of vaccine design have some limitations. For example, they could not be used to develop vaccines against microbes that do not grow *in vitro* (e.g., *Mycobacterium leper*, papilloma virus type 16 and 18). They do not provide broadly protective vaccines against pathogens with antigenic hypervariability (e.g., serogroup B meningococcus, HIV, HCV) or against pathogens with an intracellular phase, causing infections that are predominantly controlled by T cells, such as tuberculosis and malaria (6). Finally, traditional approaches of vaccine development can be very slow and time consuming, not allowing a rapid response to the need of a new vaccine, as in case of an influenza pandemic.

**Figure 1:**
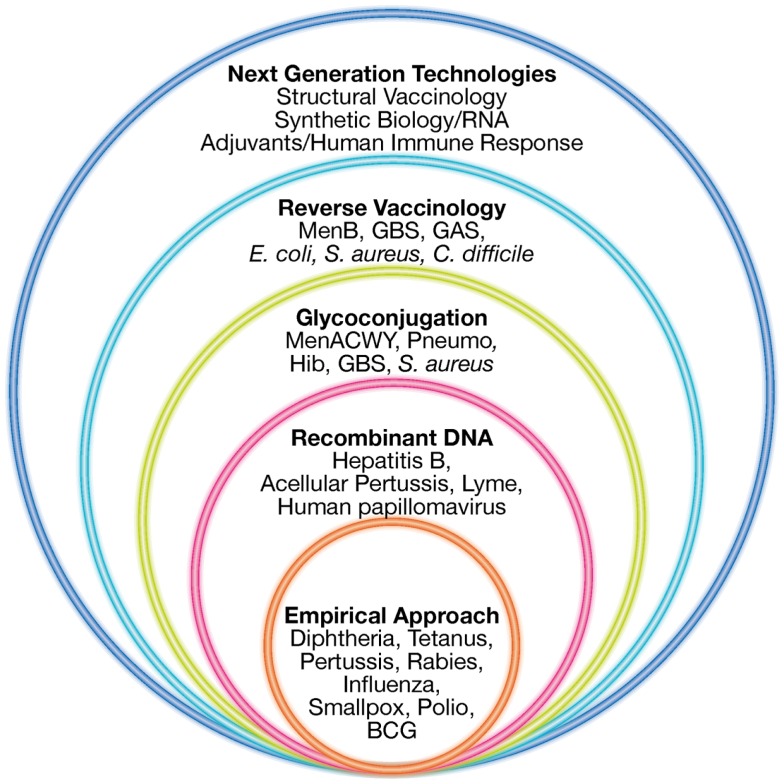
**Technologies for vaccine development**. Empirical approaches consisting mostly of killed or live-attenuated microorganisms, partially purified components of pathogens (subunit vaccines), detoxified toxins or polysaccharides have represented the starting point in vaccinology, leading to the successful elimination of many devastating diseases. During the last 30 years, several novel technologies such as recombinant DNA technology, glyconjugation, reverse vaccinology, and structural vaccinology are opening the possibility of designing new vaccines previously considered impossible to make. Adapted from Rappuoli et al. (68).

To overcome all these limitations, during the last 30 years new technologies have been applied to vaccine development. Recombinant DNA, polysaccharide chemistry, and more recently reverse vaccinology (RV), structural vaccinology, and synthetic RNA vaccines are opening up the view for the designing and development of “third generation” vaccines, previously defined as impossible to make (Figure 1).

## Polysaccharide Chemistry and Glyco-Conjugate Vaccines

One of the major immunological problems faced in the development of polysaccharide vaccines has been their low immunogenicity especially in children below 2 years of age, who represent the main target population of vaccination (7, 8). Bacterial polysaccharides are made of repeated monosaccharides linked together by glycosidic linkages. Their multiple identical antigenic epitopes cross-link multiple membrane immunoglobulins on a B cell to allow activation without the help of T cells (9). Polysaccharides cannot be processed and presented to T-helper cells and because of the lack of T-cell help, there is no germinal center reaction and the associated isotype switching, avidity maturation of the B cell receptors and induction of memory B cells.

Vaccines composed of plain bacterial polysaccharides have been introduced since the 1970s to control diseases caused by *Neisseria meningitidis, Streptococcus pneumoniae*, and *Haemophilus influenzae* type b (Hib) (10–12). However, these vaccines were poorly immunogenic in infants >2 years of age and did not appear to provide herd immunity, which is now recognized as a key element to prevent invasive bacterial infections in children. To improve immunogenicity of plain polysaccharides, they were chemically conjugated to protein carriers such as tetanus toxoid (TT), diphtheria toxoid (DT), and a non-toxic cross-reacting mutant of DT (CRM197) (13). Glyco-conjugate vaccines activate B cells via engagement of the B cell receptor following polysaccharide binding, processing of the protein carrier by polysaccharide-specific B cells, and presentation of the resulting peptides or glycopeptides in association with MHC class II molecules to T-helper cells. The MHC class II-restricted cognate interaction between B and T cells provides the costimulatory signals to B cells to start the germinal center reaction with somatic hypermutation and class-switch recombination generating B cells that will secrete high-avidity IgG antibody against the polysaccharide antigen (14, 15). As a consequence, vaccination with protein–polysaccharide conjugate vaccines is able to induce a long last immune response, with high affinity IgG antibodies and with the capacity to be boosted by subsequent immunizations (14, 16, 17). Protein–polysaccharide conjugate vaccines were introduced in the 1980s against *H. influenzae* type b (Hib) (18–20) inducing a better and persistent antibody response in all age groups. Today, different strategies to prepare conjugate vaccines can be used and effective glyco-conjugate vaccines are available for *S. pneumoniae* and the strains A, C, W, and Y of *N. meningitidis* (meningococcal meningitis) (21, 22). These vaccines are highly immunogenic and brought a huge reduction of bacterial infections in those countries that have introduced them into their immunization schedules (23–25). Although the progress made in the technology of glyco-conjugate vaccines made possible the successful control of different bacterial infections, this approach could not be applied to develop *N. meningitidis* type B (MenB) vaccine. MenB is a major cause worldwide of meningitis and sepsis, two devastating diseases that can kill children and young adults within hours (26). It is a gram-negative bacterium part of the commensal flora that colonizes the upper respiratory tract of healthy individuals. In a small proportion of cases, the bacterium can invade the host bloodstream and, after crossing the blood–brain barrier, causes meningitis (27, 28). The unsuccessful attempt of developing a MenB vaccine based on its capsular polysaccharide was largely due to the fact that it is identical to the polysialic acid present in human glycoproteins such as N-CAM. Many efforts were directed toward the development of a protein-based vaccine, all frustrated by the inconsistency of the protection data probably due to the extreme variability of the known surface proteins tested as vaccine antigens.

## Reverse Vaccinology

A major revolution in vaccine discovery is linked to the advent of genome sequencing technologies that have changed the landscape in the slowly evolving field of vaccinology. Turning point was the publication in 1995 of the genome sequence of the first living organism (29). By sequencing the genome and by determining the whole antigenic repertoire of the infectious organism, several candidate protective targets could be identified and tested for their suitability as vaccine. The method, named Reverse Vaccinology (RV), has provided a change in the perspective of vaccine design. The idea of the RV was originated to overcome the problems faced to develop an efficacious vaccine against MenB. The genome sequencing of the MenB virulent strain MC58 (30) allowed to select from the genomic data potential vaccine targets (31). The principle at the basis of the RV approach was that successful vaccine targets were proteins either exposed on the surface of the pathogen or secreted into the extracellular milieu. Starting from 2,158 encoded proteins bioinformatics analysis predicted that over 600 were either surface exposed or secreted. Of these, 350 were cloned in *Escherichia coli*, expressed and used to immunize mice. The sera of immunized animals were screened in a bactericidal assay that is known to correlate with protection. At each step candidates not satisfying quality criteria were discarded; the process led to the identification of previously unknown vaccine candidates. Through this process three protective antigens that are common to multiple MenB strains have been identified (fHbp, NadA, and NHBA) and combined with a MenB outer membrane vesicle (OMV) resulting in the first universal vaccine against MenB (32). This is the first vaccine based on RV that has recently received a positive opinion from the European Medicines Agency and has been approved with the commercial name of Bexsero^®^. Following the success of the MenB project, the RV approach has been applied to a variety of other important pathogens, such as *S. pneumoniae* (33, 34), *Streptococcus pyogenes* (35), *Chlamydia pneumonia* (36), *Chlamydia trachomatis* (37), *Streptococcus agalactiae* (38), *E. coli* (39), and *Leishmania major* (40). Thus, the genome-based RV strategy can provide innovative solutions for the design of vaccines difficult or even impossible to develop using conventional methods (41).

## Next Generation Technologies for Vaccine Design

**Figure 2:**
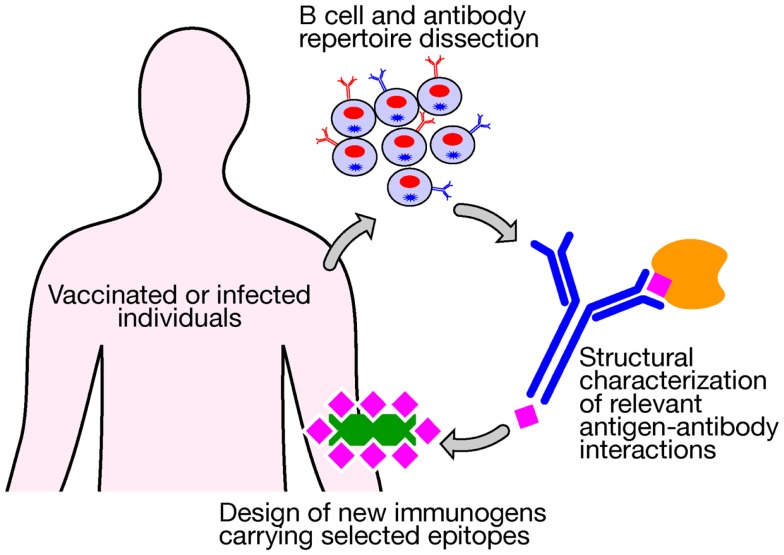
**New technologies aimed at interrogating the human B cell repertoire after vaccination or infection are providing for the first time the possibility of isolating broadly neutralizing antibodies that target relevant conserved epitopes**. A structural characterization of an antigen in complex with protective antibodies represents the launching point for the design of new immunogens bearing selected relevant protective epitopes to be used for vaccination.

Novel technologies currently under investigation represent the most valuable tools to be applied in vaccinology and could be used today for addressing the medical needs of the twenty-first century. Despite decades of efforts and investigation, satisfactory vaccines have not yet been developed against several of the most life-threatening infections, including tuberculosis, malaria, and HIV, which claim the lives of more than 4 million people worldwide each year. The high levels of variability of their antigenic proteins and the required induction of both humoral and cellular immune responses have not allowed us to use conventional vaccinology methods as successful strategies. The advent of a new approach, named structural vaccinology, could represent today a valid revolutionary alternative leading in the next years to an efficacious vaccine design. Through the combination of human immunology, structural biology, and bioinformatics knowledge, antigenic epitopes are identified based on the protein amino acid sequences and the resulting secondary and tertiary structures. The principle is based on the observation that an efficacious immune response does not require the recognition of the entire antigenic protein, but the recognition of multiple selected epitopes might be sufficient to induce protective immunity (42). Progresses in technologies aimed at interrogating the human B cell repertoire are providing for the first time the possibility of isolating broadly neutralizing antibodies targeting relevant conserved epitopes (43–45). A deeper characterization of the crystal structure of an antigen in complex with protective antibodies represents the launching point for immunogen design to select relevant epitopes from a vaccine standpoint. Once identified they can be expressed in a recombinant form and in an immunodominant fashion to be used as potent immunogens (Figure 2). Recently, the group of Kwong et al. (46) using a structure-based approach designed an immunogen for respiratory syncytial virus (RSV) that elicits higher protective responses than the postfusion form of the fusion glycoprotein, which is one of the current leading RSV vaccine candidates entering clinical trials. Importantly, highly protective responses were elicited in both mice and macaques. Structural vaccinology combined with human immunology are therefore rapidly emerging as a powerful alternative strategy for the rational design of engineered vaccines bearing multiple antigenic epitopes offering the opportunity of developing broadly effective immunity (47–49).

Another challenge in vaccinology is due to the limited capacity of the immune system to develop potent and sustained antibody responses at the extremes of age. Several studies in the last decades have shown that antigen exposure in early life results in blunted, delayed, or undetectable antibody responses to infections and immunizations (50, 51). Effective IgG responses in infants require several doses of vaccine, and to avoid a rapid wane of the titers booster doses might be required after the first year of life. At the other extreme of age, a significant decline in the capacity to induce protective antibody titers is observed in individuals of 65 years or older. The discovery of new adjuvants that help in eliciting the appropriate sustained antibody response represents a valuable strategy to overcome age-related hypo-responsiveness to vaccination. MF59 is an oil-in-water emulsion potent vaccine adjuvant licensed in more than 20 countries for more than 13 years for use in an influenza vaccine focused on elderly subjects. Recently, MF59 has been shown to be safe also in a seasonal influenza vaccine given to infants and children, providing an increase in vaccine efficacy from 43 to 89% (52, 53). However, in the last years increased knowledge of the molecular mechanisms underlying the immune response is leading to the discovery of molecules that can trigger the immune system modulating antigen-specific immunity. Monophosphoryl lipid A (MPL) is known to be a highly specific agonist of the toll-like receptor 4 (54). MPL adsorbed onto aluminum salts has been included in the formulation of the AS04-adjuvanted HPV-vaccine, the first example of a toll-like receptor agonist to be licensed as part of a vaccine for human use (55). As MPL other newly discovered innate immune receptor agonists might be ideal molecules to be exploited as adjuvants for next generation vaccines aimed at improving immunogenicity mainly in extreme ages.

Finally, together with the above described technologies used to design new protective antigens and to optimally present them to the immune system, also the new synthetic methods of vaccine production are driving the development of the twenty-first century vaccines. Nucleic acid-based vaccines represent a key advancement in combining the benefits of *in situ* expression of antigens, with the safety of inactivated and subunit vaccines. They might represent a valuable tool to overcome problems encountered when designing vaccines against pathogens that require a protective immunity mediated not only by antibodies but also by T cells. Up to now the success rate of vaccine development decreases with the decreasing ability of antibodies to confer protective immunity. A large body of literature suggests that cytotoxic T cells are important in protection from infectious diseases, such as tuberculosis, malaria, and AIDS. This would require the creation of vaccines able to induce strong T-cell responses, a weakness for existing vaccine approaches. The evidence that CD8+ T cells can control infections comes mainly from HIV. Given the failure to protect using the antibody-based gp120 vaccines (56, 57) T-cell-based vaccines have been widely tested in non-human primates (58) demonstrating to be promising. Nevertheless an HIV prime/boost vaccine based on adenovirus vector delivering T-cell epitopes failed to protect patients from disease, and had little impact on viral load (59). It is possible that this result may represent a proof-of-concept that pure T-cell vaccines are not a solution for preventive vaccines. However, there remains a need for vaccines to protect against infections for which antibodies are not sufficient or against non-infectious diseases such as cancer or autoimmunity, where also T cell immunity plays a critical role in conferring protection. DNA based vaccines showed to be very promising in animals, but in humans the magnitude of the immune response was lower than that observed with conventional vaccines. To overcome these limitations, approaches as DNA delivery by electroporation and stimulation of the immune system via the use of genetic adjuvants (i.e., *in situ* expression of immunologically active molecules encoded by the DNA vaccine) have been used in human clinical trials with encouraging preliminary results (60, 61). RNA vaccines represent a valid alternative over DNA vaccines. They are based on mRNA and self-amplifying RNA replicons that when injected intramuscularly in mice result in local production of an encoded reporter protein (62) and induction of immune responses against the encoded antigen (63). RNA vaccines have several advantages compared to DNA vaccines. RNA would eliminate the issue of possible integration of plasmid DNA into the genome of the immunized host, and it is translated directly in the cytoplasm (64). It has not been clearly elucidated the mechanisms utilized by RNA vaccines to induce an immune response, but it is likely that expression and presentation of encoded antigens follow rules similar to DNA vaccines. The efficiency and stability of RNA-based vaccines have been increased through the use of viral-particle engineered to express a heterologous antigen in place of the viral structural genes. RNA vaccines, particularly self-amplifying replicons, have therefore the potential of capturing the advantages of both DNA vaccines and viral delivery while overcoming the drawbacks of each technology. These favorable observations, supported by preclinical proof-of-concept in animal tumor models, have led mRNA vaccines into human clinical trials as immunotherapeutics in metastatic melanoma and renal cell carcinoma patients (65) showing to be able to elicit antigen-specific immune responses (both antibodies and T cells). Clinical trials have also been performed with RNA replicon vaccines packaged in viral particles encoding for cytomegalovirus (CMV) gB and pp65/IE1 proteins. The vaccine has shown to be well tolerated and immunogenic in healthy CMV seronegative volunteers, with the added value of inducing also CD8+ T-cell responses (66). The future of the RNA vaccines will rely on the formulation with new synthetic delivery systems to combine the effectiveness of live attenuated vaccines, an equal or better safety profile than plasmid DNA vaccines, and completely synthetic methods of manufacture.

Improvements in the synthetic vaccines research have provided a unique tool to rapidly respond to the need of vaccine availability in case of flu pandemics. Dormitzer et al. have developed a synthetic approach to generate vaccine viruses from sequence data (67). Starting from the available hemagglutinin (HA) and neuraminidase (NA) gene sequences, a cell-free gene assembly technique has allowed rapid, accurate gene synthesis. Viral RNA expression constructs encoding HA and NA and plasmid DNAs encoding viral backbone genes were used to transfect Madin–Darby canine kidney (MDCK) cells, qualified for vaccine manufacture. Viruses for use in vaccines were rescued from MDCK cells with increased yield of the essential vaccine antigen, HA. The implementation of synthetic vaccine seeds has demonstrated the capability of accelerating the response to influenza pandemics reducing the time required for vaccine manufacturing from months to weeks.

## Concluding Remarks

In conclusion, the last 30 years have represented a turning point in vaccinology. New technologies such as recombinant DNA, polysaccharide chemistry, and more recently RV, structural vaccinology, and synthetic RNA vaccines have greatly improved the efficiency of the vaccine-target identification, selection, and development process. Continuous progresses will be made in the twenty-first century to design new vaccines that will become the most efficient life insurance of the modern society, contributing significantly to a disease-free long life.

## Conflict of Interest Statement

The authors declare that the research was conducted in the absence of any commercial or financial relationships that could be construed as a potential conflict of interest.

## References

[1] Willis NJ Edward Jenner and the eradication of smallpox. Scott Med J (1997) 42:118–21 9507590 10.1177/003693309704200407

[2] Lakhani S Early clinical pathologists: Edward Jenner (1749-1823). J Clin Pathol (1992) 45:756–8 10.1136/jcp.45.9.756 1401201 PMC495097

[3] Kaushik DK Sehgal D Developing antibacterial vaccines in genomics and proteomics era. Scand J Immunol (2008) 67:544–52 10.1111/j.1365-3083.2008.02107.x 18397199

[4] Rappuoli R Miller HI Falkow S Medicine. The intangible value of vaccination. Science (2002) 297:937–9 10.1126/science.1075173 12169712

[5] Plotkin SA Vaccines: past, present and future. Nat Med (2005) 11:S5–11 10.1038/nm1209 15812490 PMC7095920

[6] Rappuoli R From Pasteur to genomics: progress and challenges in infectious diseases. Nat Med (2004) 10:1177–85 10.1038/nm1129 15516917 PMC7096024

[7] Makela PH Peltola H Kayhty H Jousimies H Pettay O Ruoslahti E Polysaccharide vaccines of group A *Neisseria meningtitidis* and *Haemophilus influenzae* type b: a field trial in Finland. J Infect Dis (1977) 136(Suppl):S43–50 10.1093/infdis/136.Supplement.S43 408432

[8] Peltola H Makela H Kayhty H Jousimies H Herva E Hallstrom K Clinical efficacy of meningococcus group A capsular polysaccharide vaccine in children three months to five years of age. N Engl J Med (1977) 297:686–91 10.1056/NEJM197709292971302 408682

[9] Lesinski GB Westerink MA Novel vaccine strategies to T-independent antigens. J Microbiol Methods (2001) 47:135–49 10.1016/S0167-7012(01)00290-1 11576678

[10] Conaty S Watson L Dinnes J Waugh N The effectiveness of pneumococcal polysaccharide vaccines in adults: a systematic review of observational studies and comparison with results from randomised controlled trials. Vaccine (2004) 22:3214–24 10.1016/j.vaccine.2003.08.050 15297076

[11] Lepow ML Goldschneider I Gold R Randolph M Gotschlich EC Persistence of antibody following immunization of children with groups A and C meningococcal polysaccharide vaccines. Pediatrics (1977) 60:673–80 411104

[12] Peltola H Kayhty H Virtanen M Makela PH Prevention of *Hemophilus influenzae* type b bacteremic infections with the capsular polysaccharide vaccine. N Engl J Med (1984) 310:1561–6 10.1056/NEJM198406143102404 6610125

[13] Makela PH Kayhty H Evolution of conjugate vaccines. Expert Rev Vaccines (2002) 1:399–410 10.1586/14760584.1.3.399 12901578

[14] Lai Z Schreiber JR Antigen processing of glycoconjugate vaccines; the polysaccharide portion of the pneumococcal CRM(197) conjugate vaccine co-localizes with MHC II on the antigen processing cell surface. Vaccine (2009) 27:3137–44 10.1016/j.vaccine.2009.03.064 19446183

[15] Smith KG Light A Nossal GJ Tarlinton DM The extent of affinity maturation differs between the memory and antibody-forming cell compartments in the primary immune response. EMBO J (1997) 16:2996–3006 10.1093/emboj/16.11.2996 9214617 PMC1169918

[16] Avci FY Kasper DL How bacterial carbohydrates influence the adaptive immune system. Annu Rev Immunol (2010) 28:107–30 10.1146/annurev-immunol-030409-101159 19968562

[17] Pollard AJ Perrett KP Beverley PC Maintaining protection against invasive bacteria with protein-polysaccharide conjugate vaccines. Nat Rev Immunol (2009) 9:213–20 10.1038/nri2494 19214194

[18] Black SB Shinefield HR Hiatt RA Fireman BH Efficacy of *Haemophilus influenzae* type b capsular polysaccharide vaccine. Pediatr Infect Dis J (1988) 7:149–56 10.1097/00006454-198803000-00003 3258659

[19] Eskola J Peltola H Takala AK Kayhty H Hakulinen M Karanko V Efficacy of *Haemophilus influenzae* type b polysaccharide-diphtheria toxoid conjugate vaccine in infancy. N Engl J Med (1987) 317:717–22 10.1056/NEJM198709173171201 3306379

[20] Schneerson R Barrera O Sutton A Robbins JB Preparation, characterization, and immunogenicity of *Haemophilus influenzae* type b polysaccharide-protein conjugates. J Exp Med (1980) 152:361–76 10.1084/jem.152.2.361 6967514 PMC2185954

[21] Costantino P Norelli F Giannozzi A D’Ascenzi S Bartoloni A Kaur S Size fractionation of bacterial capsular polysaccharides for their use in conjugate vaccines. Vaccine (1999) 17:1251–63 10.1016/S0264-410X(98)00348-X 10195638

[22] Lakshman R Finn A Meningococcal serogroup C conjugate vaccine. Expert Opin Biol Ther (2002) 2:87–96 10.1517/14712598.2.1.87 11772343

[23] Black S Klein NP Shah J Bedell L Karsten A Dull PM Immunogenicity and tolerability of a quadrivalent meningococcal glycoconjugate vaccine in children 2-10 years of age. Vaccine (2010) 28:657–63 10.1016/j.vaccine.2009.10.104 19895922

[24] Heath PT McVernon J The UK Hib vaccine experience. Arch Dis Child (2002) 86:396–9 10.1136/adc.86.6.396 12023165 PMC1762993

[25] Ramsay ME McVernon J Andrews NJ Heath PT Slack MP Estimating *Haemophilus influenzae* type b vaccine effectiveness in England and Wales by use of the screening method. J Infect Dis (2003) 188:481–5 10.1086/376997 12898433

[26] Harrison LH Trotter CL Ramsay ME Global epidemiology of meningococcal disease. Vaccine (2009) 27(Suppl 2):B51–63 10.1016/j.vaccine.2009.04.063 19477562

[27] Lo H Tang CM Exley RM Mechanisms of avoidance of host immunity by *Neisseria meningitidis* and its effect on vaccine development. Lancet Infect Dis (2009) 9:418–27 10.1016/S1473-3099(09)70132-X 19555901

[28] Stephens DS Biology and pathogenesis of the evolutionarily successful, obligate human bacterium *Neisseria meningitidis*. Vaccine (2009) 27(Suppl 2):B71–7 10.1016/j.vaccine.2009.04.070 19477055 PMC2712446

[29] Fleischmann RD Adams MD White O Clayton RA Kirkness EF Kerlavage AR Whole-genome random sequencing and assembly of *Haemophilus influenzae* Rd. Science (1995) 269:496–512 10.1126/science.7542800 7542800

[30] Tettelin H Saunders NJ Heidelberg J Jeffries AC Nelson KE Eisen JA Complete genome sequence of *Neisseria meningitidis* serogroup B strain MC58. Science (2000) 287:1809–15 10.1126/science.287.5459.1809 10710307

[31] Pizza M Scarlato V Masignani V Giuliani MM Arico B Comanducci M Identification of vaccine candidates against serogroup B meningococcus by whole-genome sequencing. Science (2000) 287:1816–20 10.1126/science.287.5459.1816 10710308

[32] Giuliani MM Adu-Bobie J Comanducci M Arico B Savino S Santini L A universal vaccine for serogroup B meningococcus. Proc Natl Acad Sci U S A (2006) 103:10834–9 10.1073/pnas.0603940103 16825336 PMC2047628

[33] Barocchi MA Ries J Zogaj X Hemsley C Albiger B Kanth A A pneumococcal pilus influences virulence and host inflammatory responses. Proc Natl Acad Sci U S A (2006) 103:2857–62 10.1073/pnas.0511017103 16481624 PMC1368962

[34] Gianfaldoni C Censini S Hilleringmann M Moschioni M Facciotti C Pansegrau W *Streptococcus pneumoniae* pilus subunits protect mice against lethal challenge. Infect Immun (2007) 75:1059–62 10.1128/IAI.01400-06 17145945 PMC1828493

[35] Mora M Bensi G Capo S Falugi F Zingaretti C Manetti AG Group A *Streptococcus* produce pilus-like structures containing protective antigens and Lancefield T antigens. Proc Natl Acad Sci U S A (2005) 102:15641–6 10.1073/pnas.0507808102 16223875 PMC1253647

[36] Montigiani S Falugi F Scarselli M Finco O Petracca R Galli G Genomic approach for analysis of surface proteins in *Chlamydia pneumoniae*. Infect Immun (2002) 70:368–79 10.1128/IAI.70.1.368-379.2002 11748203 PMC127649

[37] Finco O Frigimelica E Buricchi F Petracca R Galli G Faenzi E Approach to discover T- and B-cell antigens of intracellular pathogens applied to the design of *Chlamydia trachomatis* vaccines. Proc Natl Acad Sci U S A (2011) 108:9969–74 10.1073/pnas.1101756108 21628568 PMC3116399

[38] Maione D Margarit I Rinaudo CD Masignani V Mora M Scarselli M Identification of a universal Group B *Streptococcus* vaccine by multiple genome screen. Science (2005) 309:148–50 10.1126/science.1109869 15994562 PMC1351092

[39] Moriel DG Bertoldi I Spagnuolo A Marchi S Rosini R Nesta B Identification of protective and broadly conserved vaccine antigens from the genome of extraintestinal pathogenic *Escherichia coli*. Proc Natl Acad Sci U S A (2010) 107:9072–7 10.1073/pnas.0915077107 20439758 PMC2889118

[40] Metzker ML Sequencing technologies – the next generation. Nat Rev Genet (2010) 11:31–46 10.1038/nrg2626 19997069

[41] Rappuoli R Reverse vaccinology, a genome-based approach to vaccine development. Vaccine (2001) 19:2688–91 10.1016/S0264-410X(00)00554-5 11257410

[42] Scarselli M Arico B Brunelli B Savino S Di MF Palumbo E Rational design of a meningococcal antigen inducing broad protective immunity. Sci Transl Med (2011) 3:91ra62 10.1126/scitranslmed.3002234 21753121

[43] DeKosky BJ Ippolito GC Deschner RP Lavinder JJ Wine Y Rawlings BM High-throughput sequencing of the paired human immunoglobulin heavy and light chain repertoire. Nat Biotechnol (2013) 31:166–9 10.1038/nbt.2492 23334449 PMC3910347

[44] Wilson PC Andrews SF Tools to therapeutically harness the human antibody response. Nat Rev Immunol (2012) 12:709–19 10.1038/nri3285 23007571 PMC7097371

[45] Wu X Zhou T Zhu J Zhang B Georgiev I Wang C Focused evolution of HIV-1 neutralizing antibodies revealed by structures and deep sequencing. Science (2011) 333:1593–602 10.1126/science.1207532 21835983 PMC3516815

[46] McLellan JS Chen M Joyce MG Sastry M Stewart-Jones GB Yang Y Structure-based design of a fusion glycoprotein vaccine for respiratory syncytial virus. Science (2013) 342:592–8 10.1126/science.1243283 24179220 PMC4461862

[47] Barh D Misra AN Kumar A Vasco A A novel strategy of epitope design in *Neisseria gonorrhoeae*. Bioinformation (2010) 5:77–85 10.6026/97320630005077 21346868 PMC3039994

[48] Dormitzer PR Ulmer JB Rappuoli R Structure-based antigen design: a strategy for next generation vaccines. Trends Biotechnol (2008) 26:659–67 10.1016/j.tibtech.2008.08.002 18977045 PMC7114313

[49] Dormitzer PR Grandi G Rappuoli R Structural vaccinology starts to deliver. Nat Rev Microbiol (2012) 10:807–13 10.1038/nrmicro2893 23154260

[50] Adkins B Leclerc C Marshall-Clarke S Neonatal adaptive immunity comes of age. Nat Rev Immunol (2004) 4:553–64 10.1038/nri1394 15229474

[51] Siegrist CA Aspinall R B-cell responses to vaccination at the extremes of age. Nat Rev Immunol (2009) 9:185–94 10.1038/nri2508 19240757

[52] Vesikari T Groth N Karvonen A Borkowski A Pellegrini M MF59-adjuvanted influenza vaccine (FLUAD) in children: safety and immunogenicity following a second year seasonal vaccination. Vaccine (2009) 27:6291–5 10.1016/j.vaccine.2009.02.004 19840662

[53] Vesikari T Pellegrini M Karvonen A Groth N Borkowski A O’Hagan DT Enhanced immunogenicity of seasonal influenza vaccines in young children using MF59 adjuvant. Pediatr Infect Dis J (2009) 28:563–71 10.1097/INF.0b013e31819d6394 19561422

[54] Qureshi N Takayama K Ribi E Purification and structural determination of nontoxic lipid A obtained from the lipopolysaccharide of *Salmonella typhimurium*. J Biol Chem (1982) 257:11808–15 6749846

[55] Giannini SL Hanon E Moris P Van MM Morel S Dessy F Enhanced humoral and memory B cellular immunity using HPV16/18 L1 VLP vaccine formulated with the MPL/aluminium salt combination (AS04) compared to aluminium salt only. Vaccine (2006) 24:5937–49 10.1016/j.vaccine.2006.06.005 16828940

[56] Flynn NM Forthal DN Harro CD Judson FN Mayer KH Para MF Placebo-controlled phase 3 trial of a recombinant glycoprotein 120 vaccine to prevent HIV-1 infection. J Infect Dis (2005) 191:654–65 10.1086/428404 15688278

[57] Pitisuttithum P Gilbert P Gurwith M Heyward W Martin M van GF Randomized, double-blind, placebo-controlled efficacy trial of a bivalent recombinant glycoprotein 120 HIV-1 vaccine among injection drug users in Bangkok, Thailand. J Infect Dis (2006) 194:1661–71 10.1086/508748 17109337

[58] Johnston MI Fauci AS An HIV vaccine – evolving concepts. N Engl J Med (2007) 356:2073–81 10.1056/NEJMra066267 17507706

[59] Cohen J AIDS research. Promising AIDS vaccine’s failure leaves field reeling. Science (2007) 318:28–9 10.1126/science.318.5847.28 17916696

[60] Ferraro B Morrow MP Hutnick NA Shin TH Lucke CE Weiner DB Clinical applications of DNA vaccines: current progress. Clin Infect Dis (2011) 53:296–302 10.1093/cid/cir334 21765081 PMC3202319

[61] Sardesai NY Weiner DB Electroporation delivery of DNA vaccines: prospects for success. Curr Opin Immunol (2011) 23:421–9 10.1016/j.coi.2011.03.008 21530212 PMC3109217

[62] Wolff JA Malone RW Williams P Chong W Acsadi G Jani A Direct gene transfer into mouse muscle in vivo. Science (1990) 247:1465–8 10.1126/science.1690918 1690918

[63] Martinon F Krishnan S Lenzen G Magne R Gomard E Guillet JG Induction of virus-specific cytotoxic T lymphocytes in vivo by liposome-entrapped mRNA. Eur J Immunol (1993) 23:1719–22 10.1002/eji.1830230749 8325342

[64] Probst J Weide B Scheel B Pichler BJ Hoerr I Rammensee HG Spontaneous cellular uptake of exogenous messenger RNA in vivo is nucleic acid-specific, saturable and ion dependent. Gene Ther (2007) 14:1175–80 10.1038/sj.gt.3302964 17476302

[65] Kreiter S Diken M Selmi A Tureci O Sahin U Tumor vaccination using messenger RNA: prospects of a future therapy. Curr Opin Immunol (2011) 23:399–406 10.1016/j.coi.2011.03.007 21497074

[66] Bernstein DI Reap EA Katen K Watson A Smith K Norberg P Randomized, double-blind, Phase 1 trial of an alphavirus replicon vaccine for cytomegalovirus in CMV seronegative adult volunteers. Vaccine (2009) 28:484–93 10.1016/j.vaccine.2009.09.135 19857446

[67] Dormitzer PR Suphaphiphat P Gibson DG Wentworth DE Stockwell TB Algire MA Synthetic generation of influenza vaccine viruses for rapid response to pandemics. Sci Transl Med (2013) 5:185ra68 10.1126/scitranslmed.3006368 23677594

[68] Rappuoli R Mandl CW Black S De Gregorio E Vaccines for the twenty-first century society. Nat Rev Immunol (2011) 11(12):865–72 10.1038/nri3085 22051890 PMC7098427

